# Nontypeable *Haemophilus influenzae* Genetic Islands Associated with Chronic Pulmonary Infection

**DOI:** 10.1371/journal.pone.0044730

**Published:** 2012-09-06

**Authors:** Lixin Zhang, Jingping Xie, Mayuri Patel, Arsala Bakhtyar, Garth D. Ehrlich, Azad Ahmed, Josh Earl, Carl F. Marrs, Daniel Clemans, Timothy F. Murphy, Janet R. Gilsdorf

**Affiliations:** 1 Department of Epidemiology, University of Michigan School of Public Health, Ann Arbor, Michigan, United States of America; 2 Department of Pediatrics and Communicable Diseases, University of Michigan Medical School, Ann Arbor, Michigan, United States of America; 3 Center for Genomic Sciences, Allegheny-Singer Research Institute, Pittsburgh, Pennsylvania, United States of America; 4 Departments of Microbiology and Immunology, and Otolaryngology Head and Neck Surgery, Drexel University College of Medicine, Pittsburgh, Pennsylvania, United States of America; 5 Department of Biology, Eastern Michigan University, Ypsilanti, Michigan, United States of America; 6 Division of Infectious Diseases, Department of Medicine, Department of Microbiology, New York State Center of Excellence in Bioinformatics and Life Sciences, University at Buffalo, State University of New York, Buffalo, New York, United States of America; University of Louisville, United States of America

## Abstract

**Background:**

*Haemophilus influenzae* (Hi) colonizes the human respiratory tract and is an important pathogen associated with chronic obstructive pulmonary disease (COPD). Bacterial factors that interact with the human host may be important in the pathogenesis of COPD. These factors, however, have not been well defined. The overall goal of this study was to identify bacterial genetic elements with increased prevalence among *H. influenzae* strains isolated from patients with COPD compared to those isolated from the pharynges of healthy individuals.

**Methodology/Principal Findings:**

Four nontypeable *H. influenzae* (NTHi) strains, two isolated from the airways of patients with COPD and two from a healthy individual, were subjected to whole genome sequencing using 454 FLX Titanium technology. COPD strain-specific genetic islands greater than 500 bp in size were identified by *in silico* subtraction. Open reading frames residing within these islands include known Hi virulence genes such as *lic2b, hgbA, iga, hmw1* and *hmw2*, as well as genes encoding urease and other enzymes involving metabolic pathways. The distributions of seven selected genetic islands were assessed among a panel of 421 NTHi strains of both disease and commensal origins using a Library-on-a-Slide high throughput dot blot DNA hybridization procedure. Four of the seven islands screened, containing genes that encode a methyltransferase, a dehydrogenase, a urease synthesis enzyme, and a set of unknown short ORFs, respectively, were more prevalent in COPD strains than in colonizing strains with prevalence ratios ranging from 1.21 to 2.85 (p≤0.0002). Surprisingly, none of these sequences show increased prevalence among NTHi isolated from the airways of patients with cystic fibrosis.

**Conclusions/Significance:**

Our data suggest that specific bacterial genes, many involved in metabolic functions, are associated with the ability of NTHi strains to survive in the lower airways of patients with COPD.

## Introduction

The impaired lower airways in persons with chronic obstructive pulmonary disease (COPD) and cystic fibrosis (CF) are especially susceptible to bacterial colonization and infection. Non-typeable *H. influenzae* (NTHi), which lack a polysaccharide capsule, are the most commonly isolated bacteria from lower respiratory tracts of adults with COPD and acquisition of new NTHi strains is often associated with exacerbation [1], [2], [3], [4], likely because of its important role in stimulating immune responses in damaged respiratory cells and tissues leading to further damage [5], [6]. COPD is the third leading cause of death in the United States [7], and mortality rates for COPD appear to be increasing worldwide [8].

Numerous studies have demonstrated the dynamic nature of *H. influenzae* asymptomatic colonization of the human pharynx, characterized by the carriage of multiple NTHi strains at any one time [9], [10], [11], [12], [13] and apparent rapid bacterial turnover [13], [14], [15], [16]. Thus *H. influenzae* in the upper respiratory tract form a diverse pool of organisms from which organisms that infect the lower airway emerge. The lower respiratory tract, however, likely represents a different environmental niche than the upper airway and *H. influenzae* isolated from these sites show phenotypic differences. For example, in comparison to colonizing isolates from the upper airway, pulmonary isolates from the lower airway show enhanced expression of *vacJ* and *yrb* and increased serum resistance [17]. Thirty gene products, including anti-oxidant and stress-related proteins, as well as cofactor and nutrient uptake systems were produced in greater abundance by *H. influenzae* grown in human sputum from COPD patients compared to broth-grown organisms, indicating a growth adaptation of *H. influenzae* in sputum [18]. We hypothesize that among the diverse pool of *H. influenzae* colonizing the human upper respiratory tract, strains expressing specific phenotypic characteristics exhibit a fitness advantage that allows them to persist in the lower airway and contribute to the inflammatory response that leads to COPD.

Genome comparison between pathogenic and nonpathogenic strains within a species is a powerful strategy for identifying candidate genes important for bacterial pathogenesis [19], [20], [21]. *H. influenzae* is well suited for such a comparison as gene content between strains varies considerably [22], [23] and >50% of genes identified in *H. influenzae* are not found in all strains [21], [24], [25], [26]. Associations of certain NTHi genes with otitis media have been well documented and include *lic2B*, which is involved in lipooligosaccharide biosynthesis, the *hmw* genes, which encode high-molecular-weight adhesins, and the *his* operon, which is responsible for histidine biosynthesis [27], [28]. Further, HiGI2 and HiGI7, genetic islands first described in a type b strain [29], were significantly more prevalent in NTHi isolates from children with otitis media than in those from the throats of healthy children [30].

The objective in this study was to identify additional NTHi genes contributing to CODP pathogenesis. We used *in silico* whole NTHi genome subtraction to identify candidate COPD-associated gene regions, followed by population screening by DNA hybridization to identify candidate genes selectively enriched among isolates cultured from the airways of patients with COPD compared to strains from the airways of patients with cystic fibrosis (CF) or commensal strains isolated from the upper airways of healthy individuals. This analysis identified at least four genetic islands that are more associated with bronchial infections in COPD patients.

## Materials and Methods

### Bacterial strains

The bacterial strains used in this study included 421 NTHi, 25 typeable Hi, and 28 *Haemophilus haemolyticus*. All strains were initially identified as *H. influenzae* on the basis of colonial morphology during growth on chocolate agar with bacitracin, the requirement for X and V factors, porphyrin negativity, and lack of hemolysis of horse red blood cells [31], [32], [33]. These strains were further screened to confirm species designation based on the presence of *iga* and *lgtC* genes described previously [34], [35]. In this study, we defined putative *H. influenzae* strains that were positive for *iga* and *lgtC* as *H. influenzae* and those negative as *H. haemolyticus*. The value of these markers to distinguish *H. influenzae* from *H. haemolyticus* has been documented by phylogenetic analyses [34], [36], [37] and the hybridization based method used here for species discrimination was validated previously [35]. Differentiating encapsulated from nontypeable *H. influenzae* was done by detecting the *bexA* and *bexB* genes of the capsule locus by PCR, based on our published method [38].

Previously collected strains were used in this study. Of the 421 NTHi, 96 were throat isolates collected from healthy individuals [32], [39]; 101 were sputum isolates from patients with COPD collected at the University of Michigan Medical Center laboratories, or obtained from a prospective study at the Buffalo Veterans Administration Medical Center or obtained from Dr. David Hui (Chinese University of Hong Kong); 77 were isolates from patients with CF (71 sputum samples, 1 bronchioalveolar lavage sample, and 5 throat or nasopharyngeal samples) obtained from the University of Michigan Medical Center laboratories; and 147 isolates of various origins were collected from patients with clinical conditions other than COPD and CF at the University of Michigan. The 101 COPD isolates consisted of 37 isolates from patients without exacerbtion and 64 isolates from patients with exacerbation.

Additional complete or partially sequenced *H. influenzae* were used as reference strains and included Rd (ATCC 51907), 86-028NP (from Lauren Bakaletz, Ohio State University), R2866 and R2846 (from Arnold Smith, University of Washington), and PittAA, PittBB, PittCC, PittDD, PittEE, PittFF, PittGG, PittHH, PittII and PittJJ from one of the authors (GDE), and a *H. haemolyticus* type strain, ATCC 33390.

The strains were collected from many colleagues over many years under approval by the Human Use Committees at each institution and stored at their institutions. Since the strains have no identifiers attached to them, the Univ of Michigan Human Use Committee approved their use on an EXEMPT status..“IRB EXEMPTION #4 (45 CFR 46.101(b)(4)): Research involving the collection or study of existing data, documents, records, pathological specimens, or diagnostic specimens, if these sources are publicly available or if the information is recorded by the investigator in such a manner that subjects cannot be identified, directly or through identifiers linked to the subjects.”

### *H. influenzae* genome sequencing

Two COPD strains (6P18H1 and 7P49H1) and two throat strains (22-1.21, 22-4.21) were selected for initial comparative whole genomic analyses. Strains 6P18H1 and 7P49H1 were isolated from the expectorated sputum of two different adults with COPD followed in a prospective study performed at the Buffalo VA Medical Center. Based on molecular typing of strains collected from monthly cultures of sputum samples, each of these strains was initially acquired at the time of clinical evidence of an exacerbation of COPD. The acquisition of a new strain of NTHI simultaneous with the onset of symptoms of an exacerbation represents strong evidence that these strains caused exacerbations [40]. These strains were sequenced using 454 Lifesciences FLX pyrosequencing technology (454 Life Sciences) at the Center for Genomic Sciences, Allegheny-Singer Research Institute, Pittsburgh. Each genome was sequenced to a depth of 16x, or greater, and assembled into contigs using the Newbler *de novo* Assembler Software from 454 Life Sciences. The resulting numbers of contigs from the four genomes ranged from 18 to 53. The Microbial Genome Annotation Tools and Genome Annotation Pipeline from NCBI were used to predict and annotate the coding sequences (CDSs) (http://www.ncbi.nlm.nih.gov/genomes/static/Pipeline.html). The draft genomes have been deposited with GenBank and two genomes were used in an earlier analysis [25]. The accession numbers for these genome assemblies are AAZD00000000 (22.1–21), AAZJ00000000 (22.4–21), ABWV00000000 (7P49H1), and ABWW00000000 (6P18H1).

### *In silico* genome subtraction

To assemble separate contigs into a single genome sequence file, we used finished genome sequences of *H. influenzae* strains 86-028NP and Rd KW20 as references to order contigs based on sequence alignment. When contigs could not be mapped they were concatenated and added to the end of assembled genome. Both global and local sequence alignments among the four genome sequences were used to identify genomic differences between COPD strains and throat strains. Global whole genome alignments among the four genomes were construed using Progressive Mauve algorithm under alignment parameters that are appropriate for aligning closely related genomes with moderate to high amounts of genome rearrangement [41]. COPD strain-specific genomic islands greater than 500 bp were identified. These islands were further verified by local sequence alignments using BLASTn [42] by querying each island against all four genomes sequences.

### Detection of genomic islands among the bacterial collections

The presence or absence of selected genomic islands identified by the *in silico* subtraction was determined by a high-throughput dot-blot hybridization on the Library-on-a-Slide (LOS) array platform developed previously in our laboratory [35], [43], [44].

To prepare genomic island-specific DNA probes, primers hybridizing the internal regions of each island were used to amplify DNA fragments using strain 6P18H1 as the template in a standard 30 cycles PCR reaction. The primer sequences and annealing temperatures are listed in Table 1. These PCR products were purified and fluorescein-labeled using the Fluorescein-High Prime kit from Roche Applied Science (Indianapolis, IN). In addition, a DNA concentration control probe, a mixture of seven *H. influenzae* MLST gene fragments (http://Haemophilus.mlst.net/) and the coding region of *pepN*, was prepared and labeled with digoxigenin (DIG) (DIG High Prime, Roche, Indianapolis).

**Table 1:** Oligonucleotides primer sequences for PCR and probe preparations.

Targeted genomic Island		Sequence (5′–3′)	Amplicon (bp)	Annealing temperature
G1	F	GCACTCAAAGGGGCTAAAG	1046	55°C
	R	GAAGATAATACGGCGGAATACAAT		
G2	F	TCTAAATTCATCGGAGTA	570	48°C
	R	TTTTGAGGGTTATATGAATGTC		
G6	F	GTAAAAGCGTGCGTGATGGTATGG	1334	63°C
	R	CGCTGTTTGTGCCGTTGCTAAG		
G8	F	ATTGGGTTATATTTTTCTGTC	814	50°C
	R	AATCCCTTTGCTACCATCA		
G9	F	TAACTTCAACAATAGGTCGTCCAG	444	52°C
	R	TATCTTCTATTTTAACATCTAC		
G10	F	ACGCGCTTTAATTGTTTGGTAGA	1603	58°C
	R	TTTTTGGGTGATATTGTGCTTTAG		
G11	F	CAAAGATTATGGCTACCTA	616	50°C
	R	GTTATATTTTCTTACACTCTCC		

Note: Product sizes are based on 6P18H1 genome. F – forward primer; R – reverse primer.

Bacterial cells were harvested from chocolate agar plates (BBL) incubated overnight at 37°C with 5% CO2. Total genomic DNA was extracted using the GenElute Bacterial Genomic DNA kit from Sigma-Aldrich (St. Louis, MO). DNA from all strains was arrayed in duplicates onto a single nylon membrane coated slide (Vivid™ Gene Array Slides, Pall Life Sciences, Ann Arbor, MI) as described previously [35], [44], [45].

Hybridization and detection of gene probes and the analysis of the probing results have been described in detail elsewhere [35], [43], [44], [45]. Briefly, each slide was first hybridized at 65°C in PerfectHyb Plus hybridization buffer (Sigma-Aldrich) with the digoxigenin-labeled control probe, serially washed with low- and high-stringency buffers, and analyzed. The slides were then stripped, washed, and rehybridized with a fluorescein-labeled genomic island specific probe. Spotfinder v.3.1.1 and MIDAS v.2.19 were used for spot signal extraction and normalization, respectively. The ratio of the log-transformed genomic island hybridization signal to the concentration-control signal was analyzed in the software “R”. A two-component Gaussian mixture model was fitted to classify the observed intensities into positive or negative spots [35].

### Statistical analyses

Prevalence ratios were calculated as the ratio of the proportion of clinical isolates possessing the tested genomic island to the proportion of isolates in the reference group, i.e., from throats of healthy individuals. Chi-square analysis or Fisher's exact test was used to determine the significance of the differences in genomic island proportion between groups. Benjamini-Hochberg Step-Up FDR (false discovery rate)-controlling procedure was used to adjust for multiple comparisons [46]. An adjusted *p* value of ≤0.5 was considered significant. Statistical analyses were performed with SAS software (version 9.1).

## Results

### Identification of genetic islands (>500 bp) in genomic sequences of two COPD strains

As the first step in identifying *H. influenzae* candidate genes important in COPD pathogenesis, we conducted an *in silico* genome subtraction analysis between two COPD strains, 7P49H1 and 6P18H1 isolated from two different COPD patients, and two throat strains, 22.1–21 and 22.4–21 with very different multilocus sequence types isolated from one individual at two different time points [39]. With each genome sequenced to a depth of at least16x, the sizes of the draft genome assemblies obtained ranged from 1.82Mb to 1.91Mb. These sizes are comparable to those of fully finished *H. influenzae* genomes, indicating high genome sequence coverage.

**Figure 1:**
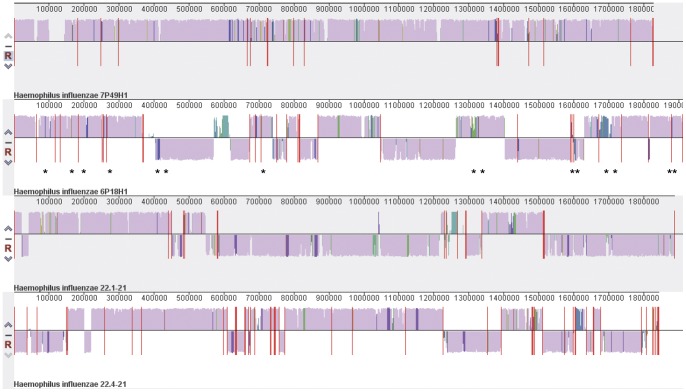
Whole genome comparison of assembled/concatenated contigs of four genomes. Two COPD strains (6P18H1 and 7P49H1) and two throat strains (22-1.21, 22-4.21) were sequence using 454FLX pyrosequencing and aligned for comparison using Mauve [38]. Regions with bars colored in mauve indicate shared sequences among four strains and regions with bars in other colors indicate sequences not shared in at least one strain. The height of the bar corresponds to average level of conservation in that region of the genome sequence. Areas that are completely white were not aligned and probably contain sequence elements specific to a particular genome. When bars lie above the center line the aligned region is in the forward orientation relative to the first genome sequence. Bars below the center line indicate regions that align in the reverse complement (inverse) orientation. The vertical red lines across center line indicate the boundary of the contigs. The stars indicated approximate positions of identified COPD specific genetic islands based on 6P18H1 genome.

Sequence analysis of these four draft genomes showed both gene content and sequence variation comparable to those seen in an earlier analysis of 12 *H. influenzae* genomes [25]. Figure 1 displays a whole genome alignment of these four roughly assembled genomes showing a common genomic backbone and genetic differences. We limited our bioinformatics analysis of the differences in the genomic contents to identify genetic islands greater than 500 bp in length so that a manageable number of genetic islands could be analyzed and to increase the probability of at least one or more coding regions within the islands. Using Progressive Mauve algorithm and BLASTn we identified 15 genetic islands (>500 bp) present in the genomes of the two NTHi strains isolated from patients with COPD and absent in two NTHi strains from healthy individuals. The sizes of these islands ranged from 628 bp to 5516 bp and totaled 33 kb in length (Table 2).

**Table 2:** CODP strain specific genetic islands identified by *in silico* subtraction.

Island	Size (bp)	Location Contig (starting bp position into contig)	No. of ORFs	Blast Match/Perdition
G1	1215	21 (22976)	1	SAM-dependent methyltransferase
G2	714	23 (267)	multiple	Potential transcriptional regulator and other small ORFs
G3	3813	23 (21413)	1	IgA-specific serine endopeptidase
G4	2818	27 (6519)	2	Carbamate kinase and hemoglobin and hemoglobin-haptoglobin binding like protein
G5	1918	48 (34610)	2	Glycosyltransferase Lic2B and undefined protein
G6	5516	48 (39066)	7	Urease operon (UreA, B, C, E, F, G, H)
G7	2705	42 (1193)	6	Transposase-like proteins and other small hypothetical proteins
G8	1617	43 (277467)	1	Aspartate-semialdehyde dehydrogenase
G9	628	43 (289778)	1	Hypothetical protein (predicted glycosyltransferase)
G10	2778	6 (65373)	1	ABC transporter ATP-binding
G11	697	6 (68252)	1	Probable TonB-dependent receptor
G12	829	7 (2916)	1	High-molecular-weight proteins (hmw locus 1)
G13	2857	17 (1290)	1	High-molecular-weight proteins (hmw locus 1)
G14	3781	20 (1)	2	High-molecular-weight proteins (hmw locus 2)
G15	962	44 (256)	1	High-molecular-weight proteins (hmw locus 2)

**Note:** Size and location are based on 6P18H1 genome. G12 and G13 are on two adjacent contigs representing two split parts of one *hmw* locus in the genome. G14 and G15 are on two adjacent contigs as well representing two split parts of the second *hmw* locus in the genome.

Each identified genetic island contained coding sequences, some representing known *H. influenzae* virulence factors while others are involved in metabolic functions whose role in pathogenesis is not clear. The glycosyl transferase gene *lic2B*, found in genetic island G5, was initially found to be important for NTHi pathogenesis based on epidemiological data [27], [47] and recently was shown to contribute to *H. influenzae* virulence by confirming serum resistance through a galactose addition to the LOS outer core [48].

Genetic island G3 contains a gene encoding an IgA protease which is different from the typical IgA protease encoding gene *iga*1 present in all *H. influenzae* and is similar to the more recently identified IgA protease encoding gene *igaB* [49], [50]. IgA1 protease activity was significantly higher in *H. influenzae* isolated from infected patients than in those isolated from throat swabs of asymptomatic carriers [51]. Recent prevalence studies showed that *igaB* is more common in strains recovered from adults with COPD than strains isolated from other clinical sources or from the throats of carriers [49], [50].

Sequences in genetic island G4 encode a hemoglobin and hemoglobin-haptoglobin binding-like protein (Hgp), important for iron acquisition and was shown to be associated with virulence in invasive infection of *H. influenzae* in animal model [52]. Genetic islands G12 to G15 all contained genes related to high-molecular-weight proteins (HMW). While labeled as four genetic islands because segments were found in four different contigs, they probably represent the two HMW loci known to be present in NTHi genomes. Islands G12 and G13 were located on contigs that mapped adjacent to each other as did islands G14 and G15. HMW mediate attachment to human epithelial cells [53] and *hmwBC*, the conserved elements in the operon, was shown to be more prevalent in NTHi isolates from the middle ears of children with otitis media than in isolates from the throats of healthy children [54].

Other genetic islands contained sequences encoding proteins involved in metabolic functions that included a SAM-dependent methyltransferase (G1), enzymes in urea metabolic pathway (G6), an aspartate-semialdehyde dehydrogenase (G8), another predicted glycosyltransferase that was different from Lic2B (G9), a likely ABC transporter (G10), and a probable TonB-dependent receptor (G11). Genetic island G2 contained multiple small open reading frames (ORFs) and its N terminus seemed to be part of a transcriptional regulator. Genetic island G7 likely originated from a transposon as it contains genes that encode transposase-like proteins and other small hypothetical proteins.

### Prevalence of selected genetic islands among *H. influenzae* from different sources

To evaluate the disease-related importance of the genetic islands identified through *in silico* subtraction, we examined by DNA hybridization their distributions among a panel of both disease-associated and commensal *H. influenzae* strains that included a random sample of 96 throat isolates from healthy individuals representing commensal NTHi, 101 disease isolates from the airways of COPD patients and 77 isolates from the airways of patients with cystic fibrosis (CF). In addition, the screening assay tested 147 NTHi strains from patients with medical conditions other than COPD and CF such as post operative infections, asthma, trauma, bronchitis and various other clinical diagnoses, isolated mostly from sputum, trachea or through bronchoalveolar lavage. In addition, a small set of typeable, encapsulated Hi and *H. haemolyticus*, a non-pathogenic organism closely related to *H. influenzae*, that also colonizes the human pharynx were included in the comparisons.

Our population screening focused on probing with genetic islands containing sequences that were previously not defined as virulence factors or only minimally studied. Thus, we excluded genetic islands G3 (IgA), G4 (Hgbs), G5 (Lic2B), and G12 to G15 (HMW) in this analysis. In addition, island G7 was also excluded because it contained mostly transposon elements, leaving seven genetic islands eligible for prevalence analysis.

The prevalence of each selected genetic island was tabulated for each of the strain groups (Table 3) and prevalence ratios (PRs) (prevalence among disease isolates/prevalence among commensal isolates) were calculated to show the relative frequencies of genetic islands in disease-associated isolates compared to throat isolates. Multiple genetic islands showed differential distributions among these strain collections. Even after adjustment for multiple comparisons, many of these differences between commensal strains and disease strains were statistically significant. Four of the 7 genetic islands, G2, G6, G8 and G10, were found to be significantly more prevalent among COPD strains compared to commensal strains. In contrast to COPD strains, only one genetic island (G1, containing a SAM-dependent methyltransferase) showed a significant difference in prevalence in CF strains compared to throat strains and it was significantly less, rather than more, frequent in CF strains than in throat strains. Compared to CF strains, NTHi isolates that originated from other clinical conditions were more similar to COPD strains in the distribution of these 7 genetic islands. The four genetic islands more prevalent in COPD strains were significantly or marginally significantly more prevalent in other clinical strains. However, the absolute prevalence of these genetics islands in those strains was lower than in COPD strains.

**Table 3:** Prevalence of seven genetic islands in *Haemophilus* isolates of different origins.

	NTHi from throats of healthy individuals (n = 96)	NTHi from COPD patients (n = 101)			Isolates from CF patients (n = 77)			Isolates from patients with clinical conditions other than COPD and CF (n = 147)			Typeable Hi (n = 25)			*H. haemolyticus* (n = 28)		
Island	No. (%)	No. (%)	PR*	*P* value	No. (%)	PR	*P* value	No. (%)	PR	*P* value	No. (%)	PR	P value	No. (%)	PR	*P* value
	with island	with island			with island			with island			with island			with island		
G1	31(32.3)	25(24.8)	0.77	0.3677	9(11.7)	0.36	0.0041	29(19.7)	0.61	0.0556	22 (88.0)	2.73	<0.0001	12(42.9)	1.33	0.4024
G2	14(14.6)	42(41.6)	2.85	0.0002	16(20.8)	1.43	0.4005	47(32.0)	2.19	0.0060	2 (8.0)	0.55	0.7247	0 (0.0)	0.12	0.3578
G6	77(80.2)	98(97.0)	1.21	0.0007	67(87.0)	1.09	0.3677	135(91.8)	1.14	0.0184	25(100)	1.25	0.0568	20(71.4)	0.89	0.4024
G8	35(36.5)	77(76.2)	2.09	<0.0001	41(53.2)	1.46	0.0556	94(63.9)	1.75	0.0002	2 (8.0)	0.22	0.0149	0 (0.0)	0.04	0.0011
G9	56(58.3)	86(85.1)	1.46	0.0002	46(59.7)	1.02	0.9100	104(70.7)	1.21	0.0848	3 (12.0)	0.21	0.0002	4 (14.3)	0.24	0.0002
G10	48(50.0)	53(52.5)	1.05	0.8223	32(41.6)	0.83	0.3930	67(45.6)	0.91	0.6031	22(88.0)	1.76	0.0019	17(60.7)	1.21	0.4024
G11	2(2.1)	8(7.9)	3.8	0.2077	1(1.2)	0.62	0.9999	5(3.4)	1.63	0.9100	9(36.0)	17.2	0.0002	1(3.6)	1.71	0.9999

* PR  =  prevalence ratios.

Note: P values were adjusted by Benjamini-Hochberg Step-Up FDR procedure to account multiple comparisons.

Although only a small number of typeable Hi and *H. haemolyticus* strains were included in the screening, the distribution of the genetic islands in these two groups showed an interesting contrast to NTHi. In general, island G1 was more and islands G2, G8 and G9 were less frequently found in typeable Hi and *H. haemolyticus* strains than in NTHi strains of all sources.

### Distribution of genetic islands between COPD isolates from patients with and without exacerbation

Other investigators, Fernaays et al, have hypothesized that differences among strains of *H. influenzae* contributed to different clinical presentations in COPD-associated infection [49]. Our COPD collection consisted of 64 strains isolated from patients with exacerbation and 37 strains isolated from patients without exacerbation. We performed a stratified comparison to test whether selected genetic islands might be associated with exacerbation (Table 4). The prevalences of six of seven genetic islands were similar (PRs close to 1) in the two groups. While PR for island G11 is 4.04, its absolute prevalence in both groups was very low and not statistically different.

**Table 4:** Prevalence of seven genetic islands in COPD NTHi isolates from patients with and without exacerbations.

Island	Isolates from COPD patients without exacerbations (n = 37)	Isolates from COPD patients with exacerbations (n = 64)	PR*	*P* value
G1	10(27.0)	15(25.8)	0.87	0.6871
G2	16(43.2)	26(40.6)	0.94	0.797
G6	37(100)	61(95.3)	0.97	0.3714
G8	28(75.7)	49(76.6)	1.01	0.9196
G9	33(89.2)	53(82.8)	0.95	0.5417
G10	18(48.6)	35(54.7)	1.12	0.5582
G11	1(2.7)	7(10.9)	4.04	0.2713

* PR  =  prevalence ratio.

## Discussion

While bacteria often utilize complex regulatory systems to alter gene expression in response to environmental changes, *H. influenzae*, with a small yet highly variable genome and a niche limited to the human respiratory tract, also appear to rely on natural selection and clonal expansion of selected strains to survive in varying environments [55]. This study exploited the process of natural selection that occurs as NTHi evolve in the human host, and uses epidemiologic analyses to identify specific *H. influenzae* genes that have been disproportionally preserved, and thus more critical to survival and disease initiation, among isolates cultured from patients with COPD compared to commensal NTHi isolated from throats of healthy individuals. Previously, we used a similar strategy that successfully identified NTHi genes associated with otitis media in which subtractive genomic hybridization was used to generate candidate sequences [27], [47]. In this study, we took advantage of next generation DNA sequencing technology to generate whole genome sequences used in *in silico* genome subtraction to generate candidate COPD-specific sequences. Among 15 genetic islands found initially, seven–those that didn't possess previously known Hi virulence factors–were subjected to population prevalence analyses utilizing a panel of 421 NTHi to identify genes significantly more prevalent among COPD strains than commensal strains. Four of these seven genetic islands (G2, G6 G8, and G9) were found to be significantly associated with NTHi isolated from the airways of COPD patients.

The genetic island G2 had the strongest association with COPD strains compared to throat strains with a prevalence ratio of 2.85. The potential function, however, of the G2 was difficult to infer from its sequence. The 714 bp fragments contained multiple ORFs no greater than 51 amino acids in size. This sequence could contain short sequences involved in gene regulation or encode small peptides. G2 was found to be highly similar to a DNA fragment 183UM identified in an early study that attempted to identify genes associated with exacerbations of COPD [49]. In that study, an alternative hypothesis was put forward that the 183UM was gained during a genetic integration and, in fact, is a marker for the acquisition of a novel IgA protease gene (*igaB*) in the genomic region. A further blast search against a protein database using all possible translated ORFs indicated that a portion of the G2 sequences have a moderate degree of similarity to insertion sequence family proteins, potentially supporting its role in gene insertion. Further studies are still needed to evaluate whether sequences on this genetic island are transcribed and have a direct role in the function of Hi pathogenesis. In addition, while 183UM was found to be associated with exacerbation in that early study we did not find a differential distribution of G2 in exacerbation and non-exacerbation COPD strains. In Fernaays study, 49.2% of exacerbation COPD isolates were positive for 183UM compared to 37% of COPD isolates not associated with exacerbation. Our COPD collection contained a subset of strains from the Fernaays study. When we limited our analysis to this subset, we found 50% exacerbation isolates were positive for G2 compared to 39% non-exacerbation isolates, almost identical to the Fernaays' finding. However, four of these strains did not meet our study definition of typical NTHi (positive for both *iga* and *lgtC* by hybridization). Once these four strains were removed from the analysis, the prevalence became almost the same for both groups. In fact, none of the genetic islands screened were significantly associated with exacerbation in our univariate analysis. As additional genes are screened we plan to perform multivariate analyses to identify sets of combined genes that differentiate exacerbation and non-exacerbation strains similar to the analysis performed in the Fernaays study [49].

Island G6 contains the entire urease operon. Urease is a nickel metalloenzyme that catalyzes urea into carbon dioxide and ammonia, generates nitrogen for bacterial growth, and allows bacteria to survive in acid environments [56]. In other bacteria, increased urease expression occurs in nitrogen-limited environments, in acidic environments, and by urea induction. Thus, urease production may be beneficial for NTHi survival and infection of the chronically inflamed airways of COPD patients while simultaneously damaging the respiratory epithelium, either by increasing the local pH or by direct ammonia toxicity. Genetic island G8, predicted to encode aspartate-semialdehyde dehydrogenase (Asd), was also strongly associated with COPD strains. Asd forms an early branch point in the metabolic pathway forming lysine, methionine, leucine, and isoleucine from aspartate and generates diaminopimelate (DAP), an essential component of the Gram-negative bacterial peptidoglycan [57]. To maintain membrane integrity *asd* auxotrophs require diamimopimelic acid (DAP) [58], which may be more abundant in the pharynx than in airways of COPD patients.

Island G9 encodes a predicted glycosyltransferase that is iron and heme inducible [59]. The neighboring genes HI1384 and HI1385 encode ferritin subunits which form a macromolecular structure that stores and detoxifies Fe when cellular levels become elevated [60]. The functional relationship of the glycosyltransferase to the ferritin is unknown but human ferritin is glycosylated [61], and thus, the bacterial ortholog may also require glycosylation for function and may in turn be important in pathogenesis.

The distribution of genetic islands among NTHi strains isolated from patients with clinical conditions other than COPD and CF mirrored that seen in COPD isolates but prevalences of these islands were comparably lower. Three of the four COPD-associated islands were also significantly more prevalent in those strains than in throat strains. Given that this collection was a mixture of strains from different sources and likely contained some lower airway NTHi pathogens with different growth requirements from those required for survival in the airways of COPD patients, we would expect lower PRs for these genetic islands when compared to that in the well-defined COPD collection.

One surprising finding was that the distribution of the genetic islands among CF strains differed from the distribution among COPD strains. Island G1 showed a negative association with CF strains, i.e. it was significantly less prevalent (PR = 0.36) in CF strains compared to throat strains while it was similarly distributed among COPD and commensal strains. G1 encodes a type of S-adenosyl-L-methionine (AdoMet)-dependent methyltransferase (MTases), enzymes that usually transfer methyl groups to compounds on substrates such as nucleic acids, proteins, and many small molecules [62] and alter the targeting and timing of gene expression and activity of certain enzymes [63]. The genetic island frequency data indicated that NTHi factors important in bronchial infection in CF patients were different than in COPD patients.

Bronchial infections in patients with COPD and CF share important clinical and pathogenic features, including long term chronicity with episodic exacerbations characterized by increased dyspnea, increased sputum volume and increased sputum purulence; progressive cycles of chronic lung inflammation and infection; and abnormal structural remodeling of the lower airways as a result of chronic inflammation and infection. The primary pathogenesis of these two conditions, however, is different. CF is an inherited disorder characterized by mutation of the cystic fibrosis transmembrane regulator (CFTR), an adenosine triphosphate-dependent chloride channel that leads to exocrine gland dysfunction. Resulting dehydrated respiratory secretions decrease normal mucociliary clearance of bacteria and facilitate chronic infection and inflammation that lead to progressive suppurative obstructive lung disease. COPD is an acquired disorder caused by airway damage, primarily from smoking, with resultant impaired bacterial clearance that leads to inflammation, chronic infection and end-airway obstruction.

The microbial components of chronic infection in patients with COPD and cystic fibrosis vary with the stage of the infection. Among patients with cystic fibrosis, *Staphylococcus aureus* and *H. influenzae* predominate in young children whereas older children and adults exhibit chronic infection with *Pseudomonas aeruginosa*, *Stenotrophomonas maltophilia* and *Burkholderia cepacia* [64]. Irrespective of the stage of COPD, *H. influenzae, Streptococcus pneumoniae*, and *Moraxella catarrhalis* are the most commonly isolated pathogens from the lower respiratory tract [40]. The explanation for the differences in the flora of these two seemingly similar disease processes remains unclear, but may rest with differences in the local milieu–nutritional, chemical, or physical–in the airways. Thus, different environmental conditions, either host or microbial community driven, in the airways of patients with COPD or cystic fibrosis may explain the differences in prevalence of specific *H. influenzae* genes as seen in this study, i.e. possession of certain genes and expression of their gene products may predispose different strains of *H. influenzae* to successful chronic infection in patients with cystic fibrosis or COPD.

Another observation was the relatively or extremely lower frequencies of those COPD associated genetic islands (G2, G6, G8, and G9) in *H. haemolyticus*. Since *H. haemolyticus* is generally considered to be non-pathogenic and does not cause disease or live in normally sterile sites [27], [36], such findings could be taken as an additional evidence to support the hypothesis that COPD-associated genetic islands might be important in NTHi pathogenesis.

In summary, this study used a molecular epidemiologic approach that combined *in silico* subtraction and population prevalence analysis to identify NTHi genes associated with lower airway infections. The results showed that several genetic sequences were associated with infections in COPD. Future studies will be directed to screen additional sequences and analyze the joint effects of these sequences epidemiologically. In addition, functional studies will be needed to elucidate the mechanisms by which these identified genes contribute to the pathogenesis.
